# Development of a new testing equipment that combines the working principles of both the split Hopkinson bar and the drop weight testers

**DOI:** 10.1186/s40064-016-2770-8

**Published:** 2016-07-22

**Authors:** Rateb Adas, Majed Haiba

**Affiliations:** Damascus University, Damascus, Syrian Arab Republic

**Keywords:** High strain rate testing, Tensile testing, Dynamic testing, Tensile testing equipment, Strain rate histories

## Abstract

In the current work, a new high strain rate tensile testing equipment is proposed. The equipment uses a pendulum device to generate an impact load and a three-bar mechanism to bring that load to act upon a specially designed specimen. As the standard impact testing apparatus uses pendulum device and the well-known SHB high strain rate tester adopts the above-mentioned mechanism, the introduced equipment can be dealt with as an impact apparatus in which the base that supports the V-shape specimen is replaced with the three-bar configuration that the traditional SHB uses. In order to demonstrate the applicability of the new tester, virtual design tools were used to determine the most appropriate configuration for it. Then, a detailed design was created, and a full-scale prototype was produced, calibrated, instrumented and tested. The obtained results demonstrate that the new tester is capable of axially straining steel specimens up to failure at a maximum rate of about 250 s^−1^, which is reasonable when compared with a more established high strain rate testers.

## Background

**Fig. 1:**
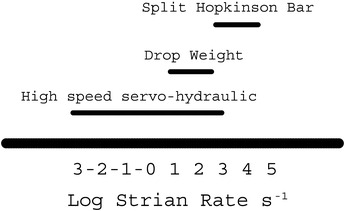
Ranges of strain rates covered by dynamic tensile testing systems (Xiao 2008)

**Fig. 2:**
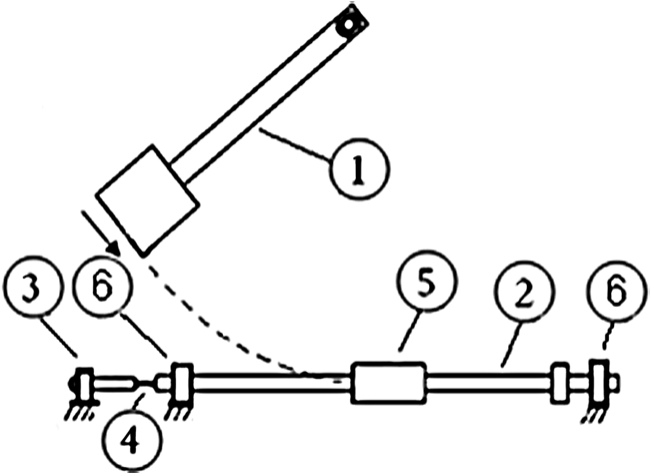
A sketch of the proposed tensile testing machine

Due to the need for optimized products, different uniaxial tensile testing techniques have been introduced to generate data under dynamic conditions. In this context, servo-hydraulic (SH) (Boyce and Crenshaw 2005), split Hopkinson bar (SHB) (Ogawa 1984) and drop weight (DW) (Chan 2009) are some of the most popular testing systems. As shown in Fig. 1, dynamic testing systems are classified based on the achievable strain rate $\dot{\varepsilon }$. According to what is shown in the figure, each of the above-mentioned systems serves for a specific range of $\dot{\varepsilon }$ and consequently fits for a specific set of applications. For automotive industry there is a clear need to use either a high-speed SH or a DW testing systems as the loading speed associated with this industry corresponds to 0.01 $\left\langle {\dot{\varepsilon }} \right\rangle$ 500 s^−1^ (Xiao 2008). Due to lack of access to high-speed SH testers, a growing interest in the DW technology was noticed (Chan 2009; Li and Liu 2009; Mott et al. 2007; Ferrini and Khachonkitkosol 2015). In spite of that, reports of work based on that technology for dynamic tensile tests are relatively scarce due to limitations and complications associated with it (Chan 2009) (“Literature review of the currently available DW tensile testers” section presents some of these shortcomings). Thus, the feasibility of modifying that technology for less complication and easier operation needs to be explored. Within this framework, the current research presents a new design that attempts to overcome some of the challenges associated with the use of the currently available DW tensile testers. As shown in Fig. 2, the working principle of the proposed tester implements a pendulum device to deliver an impact load to one end of the specimen, which is mounted between the incident and transmitter bars. A striker tube is impacting the end of the incident bar so that a stress wave is created and propagated through the incident bar, specimen and the transmitter bar. Based on the propagation theory, the stress and strain in the specimen can be determined by the strain histories measured using strain gauges which are attached to the bars. Obviously, perfect alignment of the components that the load passes through is essential to ensure pure tensile loading along the gauge section of the specimen.

## Literature review of the currently available DW tensile testers

**Fig. 3:**
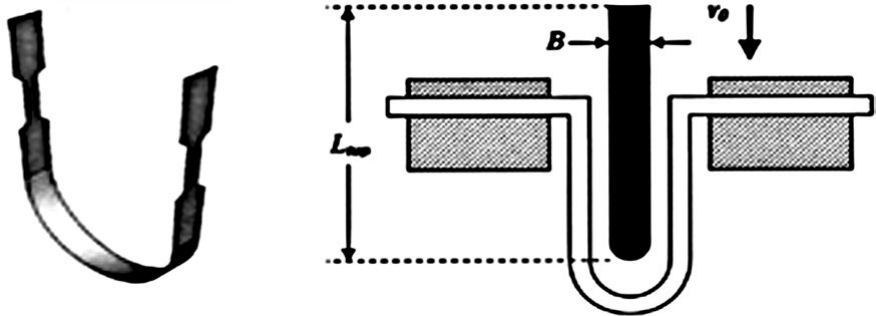
The tensile testing specimen considered by (Chan 2009)

The Droop Tower Instrument (DTI) is a high strain rate compression tester in which a weight is suspended at a height and dropped onto a specimen, and a force sensor, “which is attached to the bottom of the weight”, is used to take strain readings. Obviously, a modified form of the above setup is needed if tensile tests are to be accounted for using a traditional DTI. Within this context, a setup that involves two-dog bone specimens with grip sections at two ends and a curved connecting part was considered by (Chan 2009). As shown in Fig. 3, the dropped weight strikes the center of the specimen during the test, creating tension along the vertically aligned dog bone sections of the sheet.

**Fig. 4:**
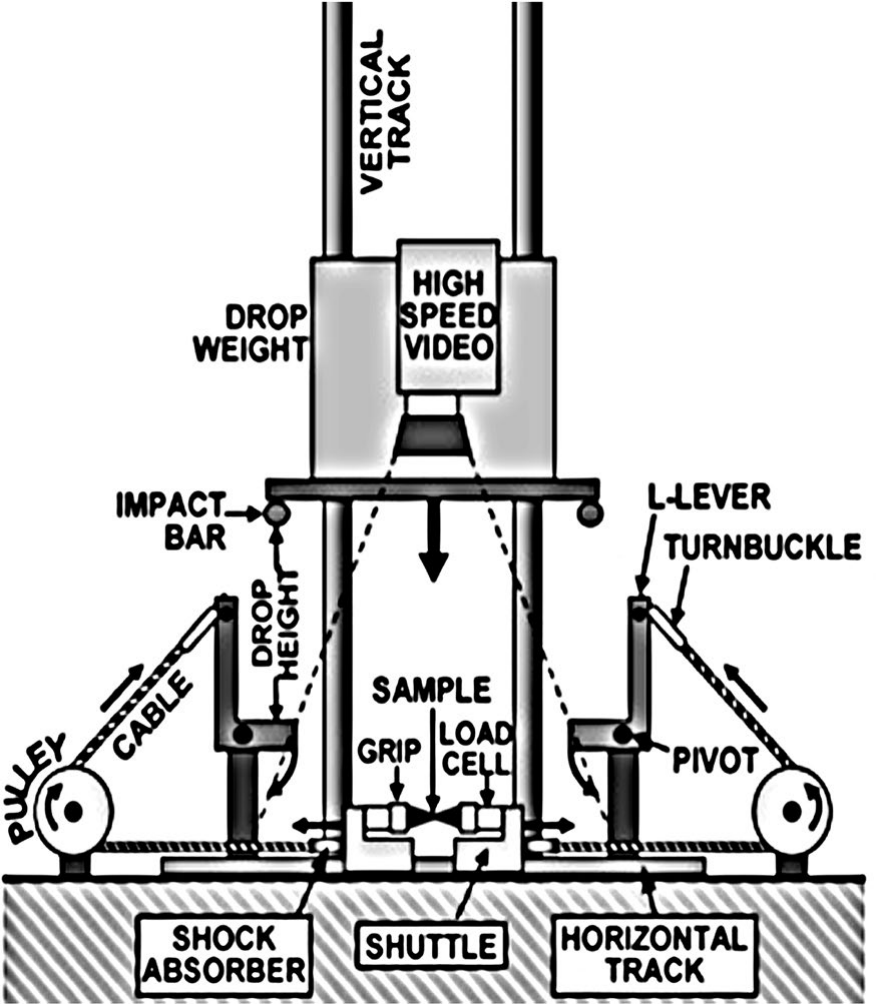
A modified design of the tensile testing DTI (Mott et al. 2007)

Clearly, the above-mentioned tensile testing configuration is problematical as bending wave is sent through the specimen, which would create an oscillation, and would also add noise to strain measurements. Obviously, replacing the two-dog bone specimen with a simpler one improves the quality of the measured stain histories, but requires a modified design of the DTI. Within this context, a modified design of the traditional DTI, which is capable of measuring the tensile response of materials, was developed by Mott et al. (2007). As shown in Fig. 4, the device uses a 100 kg drop weight which is raised on a vertical track to a given height and then released. Attached to the bottom of the weight are two round impact bars. These bars engage L levers, which pivot about bearings as the drop weight falls, to pull attached cables. The cables pass around pulleys and are attached to shuttles, which are in turn caused to move in opposite directions on linear bearings on a horizontal track. The tensile force is measured by load cells at each end of the sample, and strain in the specimen is determined by the change in length between marks at either end of the test section. Evidently, Mott’s design of the DW tensile tester uses simple shaped axially loaded specimens, but implements flexible elements, which requires distinct treatment when analyzing the recorded strain histories, and adopts a very complicated mechanism, which is reflecting in a negative manner when considering machine calibration, efficiency and expenses.

## CAD modeling and design development

The following set of requirements and constraints were taken into account during the design phases of the proposed tester:

1. Loading steel specimens up to failure at maximum strain rate of 300 s^−1^.
2. Respecting the recommended testing practices, specimen geometry, clamping method, measurement devices, and data processing methods, as stated in Borsutzki et al. (2005).
3. Minimization in size, ease of specimen mounting and dismounting and efficiency in energy management.
4. Axiality of specimen loading and centricity between the components that the impact energy basses through.

**Fig. 5:**
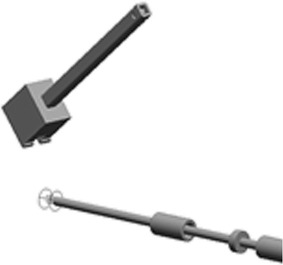
A kinetic model of the initial design of the proposed tester

In doing so, the model, which is shown in Fig. 5, was initially created using a Multi-Body System (MBS) software (Garcia). As illustrated in the figure, that model includes the following components:

- Rigid elements to represent the pendulum device (the hammer), the incident bar, the transmitter bar and the striker tube.
- A revolute joint to account for the pendulum motion of the hammer.
- A sliding joint to account for the motion of the Striker tube along the incident bar.
- Another sliding joint to account for the possible longitudinal motion between the incident bar and the two linear supports which hold it.
- A non-linear spring to stand for the specimen (Shames 1992). A low strain rate tensile test of an aluminum specimen was conducted and the non-linear stiffness of the spring was calculated using the obtained load–deflection data.

**Fig. 6:**
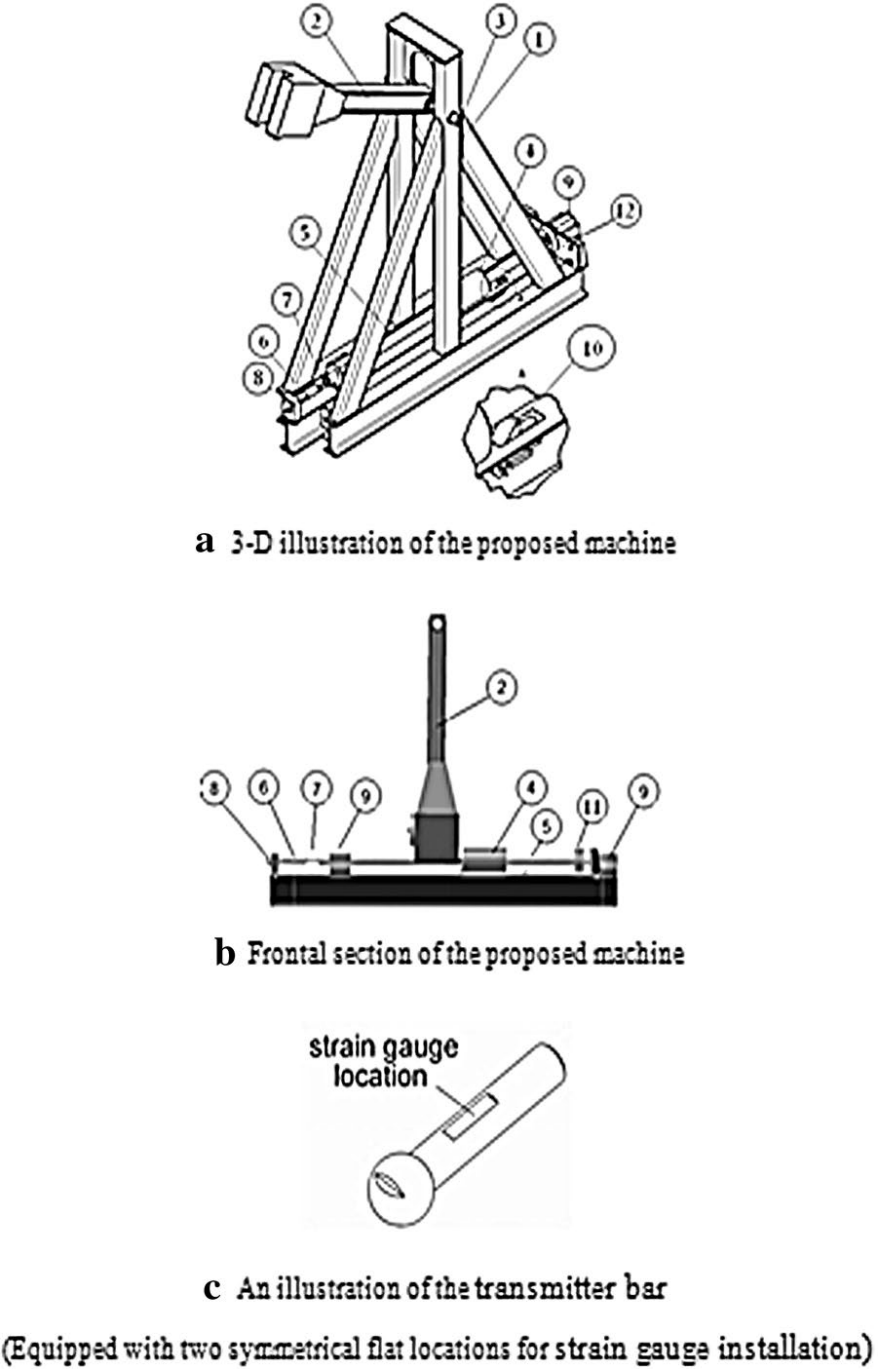
**a** 3-D illustration of the proposed machine. **b** Frontal section of the proposed machine. **c** An illustration of the transmitter bar, (equipped with two symmetrical flat locations for strain gauge installation)

Based on the results obtained from the above modeling, the design of the proposed tester was finalized via an iteration process that includes modifications, obtaining results and evaluation steps, in which all design variations were explored. Figure 6a–c illustrates the final design of the tester. This design includes the following components: (1) steel structure, (2) hammer, (3) axis of rotation, (4) striker tube, (5) incident bar, (6) transmitter bar with spherical end, (7) specimen, (8) transmitter bar support with spherical hole, (9) incident bar supports, (10) non-return lock, (11) extension of incident bar, (12) baffle fitted with a rubber damper.

In the above design, and as recommended by Borsutzki et al. **(**2005**)**, force history *F*(*t*), (required to evaluate the stress variation acting on the specimen), is calculated using Eq. (1), in which:

- $\varepsilon_{e} (t)$ is an elastic strain history (measured using a strain gauge attached to the transmitter bar, as illustrated in Fig. 6).
- *E* is the elastic moduli of the transmitter bar material.
- *A* is the cross section area of the transmitter bar (measured at the strain gauge locations)
  1 $$F\left( t \right) = E \cdot \varepsilon_{e} \left( t \right) \cdot A$$

## The structural modeling of the proposed tester

To carry out the structural study of the proposed tester, a finite element model was created using ANSYS software. The created model, which represents one-half of the structure, consists of 114,730 elements of the type “Solide45”. For constraining, symmetrical boundary conditions, to represent the missing half of the structure, and contact elements and nodal constraints, to represent the machine-ground interface, were considered. For loading, the relevant load histories, which were estimated from the abovementioned MBS simulation, were applied as nodal loads in three locations, (1) the hammer articulation, (2) the baffle, and (3) the spherical joint support. Solving the created model and reviewing the obtained results (stress, strain, and displacement contours and histories) enables the following conclusions to be drawn:

- The structure is very stiff as the maximum deflection does not exceed 5 μm.
- The structure has a minimum safety factor of about 13, as the maximum value of the equivalent stress equals 20.7 MPa.

## Producing, installing and preliminary testing of the tester

**Fig. 7:**
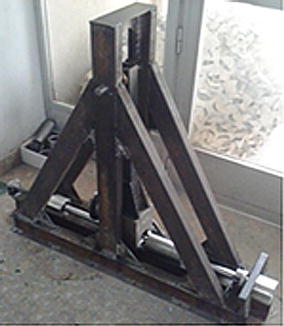
An illustration of the produced tester

**Fig. 8:**
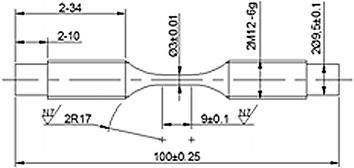
The designated design of the implemented specimens
- For preliminary testing, the specimen was firmly installed be threading it into the corresponding threaded holes of the incident and transmitter bars, respectively. The hammer was then raised up to the highest possible position and freed to hit the striker tube which moves and hits the extension of incident bar, causing the specimen to quickly extend up to failure. Due to the satisfactory performance noticed during the stage of the initial testing, it was decided to accept the designated design of the specimen and move ahead to produce and test twenty new steel specimens.

During the subsequent phases of the work, the standard and non-standard components of the tester were respectively purchased and manufactured, they then assembled and calibrated in order to account for the above-mentioned axiality and centricity requirements. The obtained hardware, which is shown in Fig. 7, was installed over a solid floor and then subjected to preliminary tests. In doing so, the following considerations were taken into account:

- Components, which are subjected to impact loading, were thermally hardened, up to 55HRC, in order to eliminate energy losses due local distortions.
- The spherical surfaces of the transmitter bar and the corresponding support were subjected to set of special treatments (hardening, polishing and greasing) in order to improve the performance of interface between them. This was essential to assure pure axial loading of specimen when loaded.
- Components, which moves relative to each other’s, were equipped with linear bearings in order to eliminate energy losses due friction.
- The three supports, which hold the bars, were accurately aligned using a standard 30 mm diameter chrome rod, produced by Bosch Rexroth Corp. An identical rod was also used to produce the bars (the raw material which was used to produce the bars is a standard 30 mm chrome rod, produced by Bosch Rexroth Corp).
- The non-return lock was calibrated in a way that maintains the kinetic energy of the striker tube when passes through it, while preventing the retreat of that tube towards the tested specimen after hitting the extension of the incident bar.
- Twelve M10 × 100 steel screws were used to rigidly install the tester over a solid floor.
- One steel specimen was manufactured according to the details shown in Fig. 8. Obviously, the designated design of the specimen is simple, easy to be mounted and dismounted and similar to what the SHB tester uses. Moreover, it respects the related recommendations, as specified in Borsutzki et al. **(**2005) and in ASTM D1822.

## Strain rate determination

The strain rate history $\dot{\varepsilon }\left( t \right),$ which is associated with a specific tensile test, is usually calculated using the formula (Borsutzki et al. 2005):

2 $$\dot{\varepsilon }\left( t \right) = \frac{dl}{l \cdot dt}$$

in which *l* is the parallel length of the specimen; *l* = 9 mm as shown in Fig. 8. $\frac{dl}{dt}$ is the extension history of the tested specimen.

Then, the determination of $\dot{\varepsilon }\left( t \right)$ for a specific tensile test requires accurate measurement of the extension history.

**Fig. 9:**
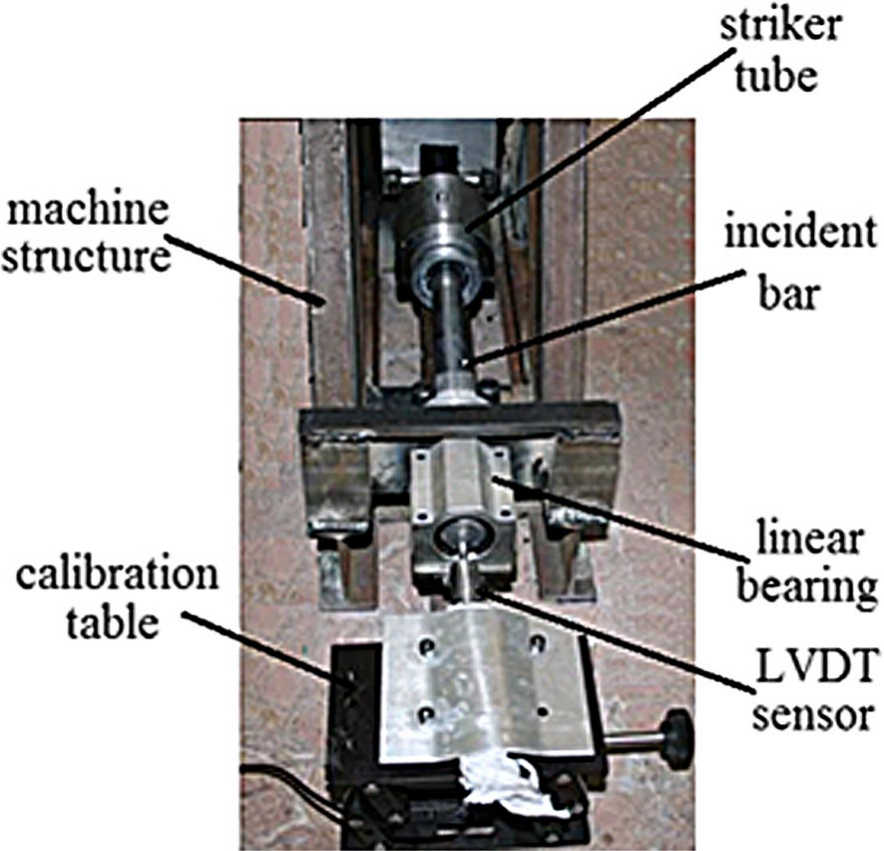
An illustration of the calibrated sensor

In the current research, it was assumed that the extension history of the loaded specimen is identical to the motion history of the incident bar, and that assumption was accepted due to the huge rigidity of the bar when compared with that of the specimen (Borsutzki et al. 2005). Thus, an acquisition system, which involves LVDT sensor, processing unit and an industrial computer, was used to record the longitudinal motion of the incident bar, when it moves due to specimen elongation and then failure. As shown in Fig. 9, a calibration table was used to accurately locate and rigidly install the sensor. In doing so, the instructions, which are listed in the user manual of the sensor, were carefully respected.

## Processing of the recorded histories

**Fig. 10:**
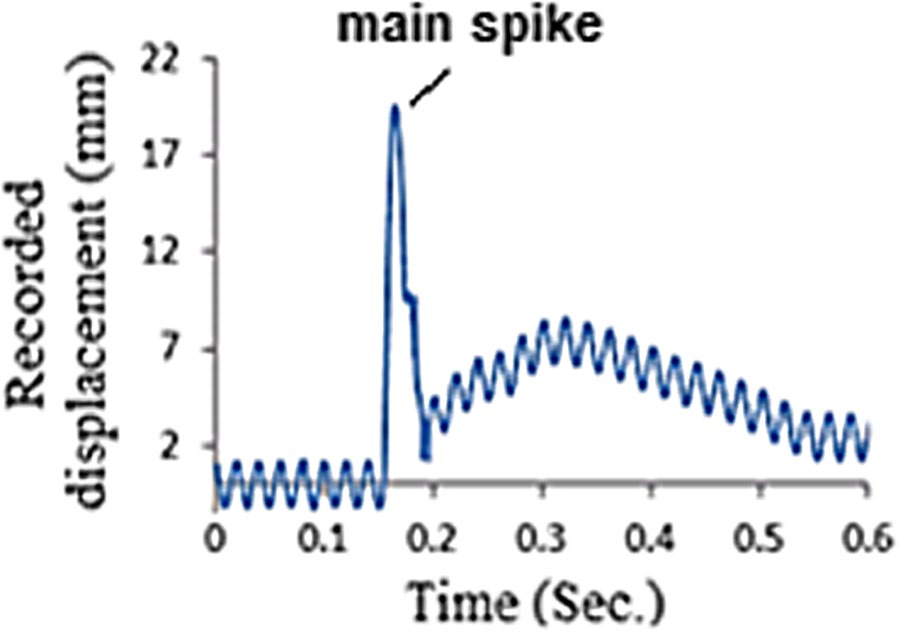
The first recorded motion history

**Fig. 11:**
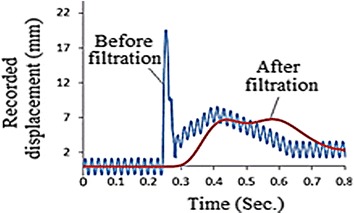
A comparison between the histories before and after the low-pass filtration

Out of the twenty specimens, one arbitrary selected sample was mounted and then tested while operating the data acquisition system. The recorded motion history of the incident bar is shown in Fig. 10. Clearly, the obtained history needs processing, as inconvenient components and noises due to the 50 Hz electrical interference superimpose the useful data (Borsutzki et al. 2005). In an attempt to overcome this matter, several standard filtration algorithms were implemented without satisfactory results. Within this context, Fig. 11 presents the results obtained using a low-pass filter. Unfortunately, the use of the low-pass filtration process was disappointed as it eliminates the main spike, which represents the most important part of the history.

For more efficient processing of the history, a modification technique that involves the following steps was adopted, as recommended by Borsutzki et al. **(**2005):

1. To eliminate the noise signal, an inverse of that signal was generated and added to the history, as illustrated in Fig. 12b.

**Fig. 12:**
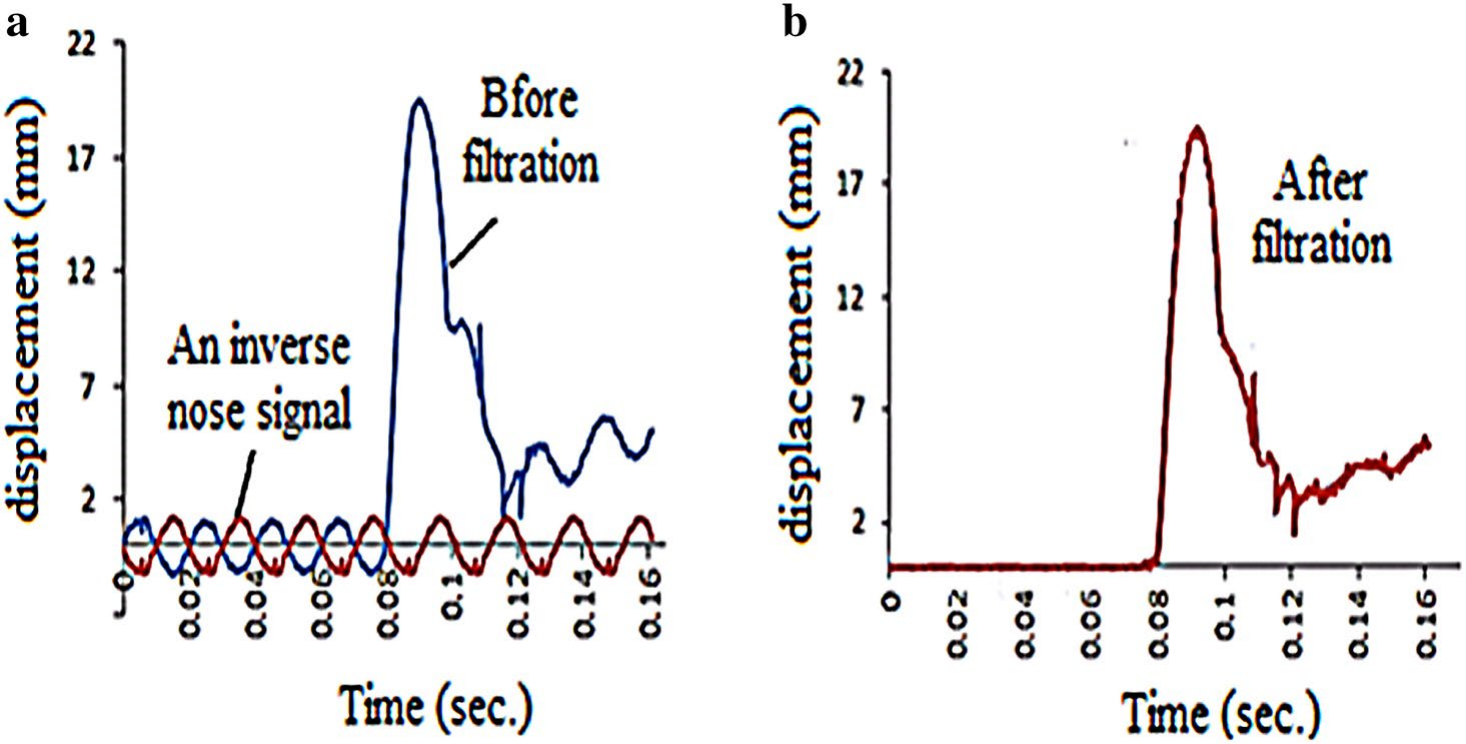
**a** History after noise filtration. **b** History before noise filtration
2. To eliminate the inconvenient components of the history the two following processes were considered:
  - Zero displacements were eliminated from the after filtration history.
  - Displacements, which were bigger than 2.1 mm, were eliminated from the history, as the 2.1 mm limit matched the measured elongation of the broken specimen; see Fig. 13 which illustrates the above-mentioned processing.

**Fig. 13:**
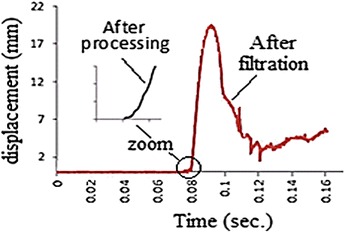
The final state of the displacement history

**Fig. 14:**
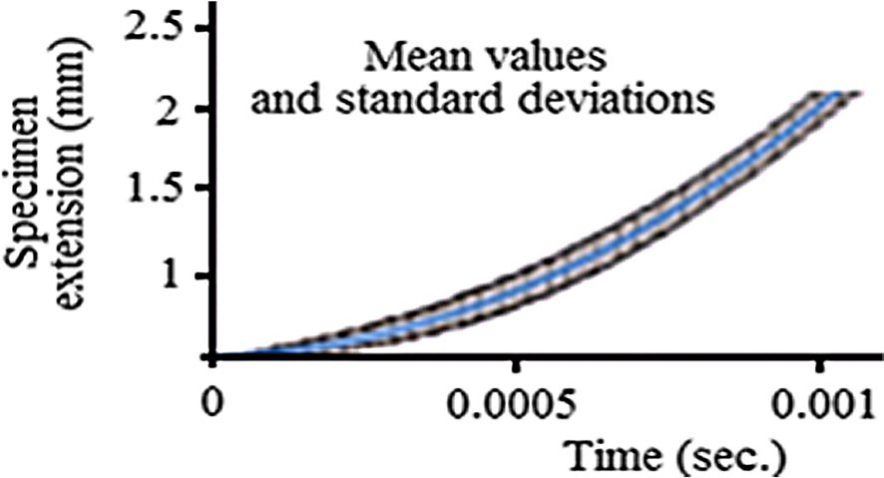
The statistical presentation of the final results

For the statistical validation of the obtained results, the remaining nineteen specimens were tested and the obtained histories were modified using the above-mentioned steps, and then mean values and standard deviations were calculated; see Fig. 14 for the graphical presentation of the final results and Table 1 for samples of the obtained values.

**Table 1:** Samples of the calculated extension results

Time (s)	Mean value of extension (mm)	Standard deviation
0.0004	0.228146088	0.081413743
0.0006	0.476465198	0.097867097
0.0008	0.821725717	0.099193456
0.001	1.269510668	0.097635866
0.00145	2.680800085	0.239405582

**Fig. 15:**
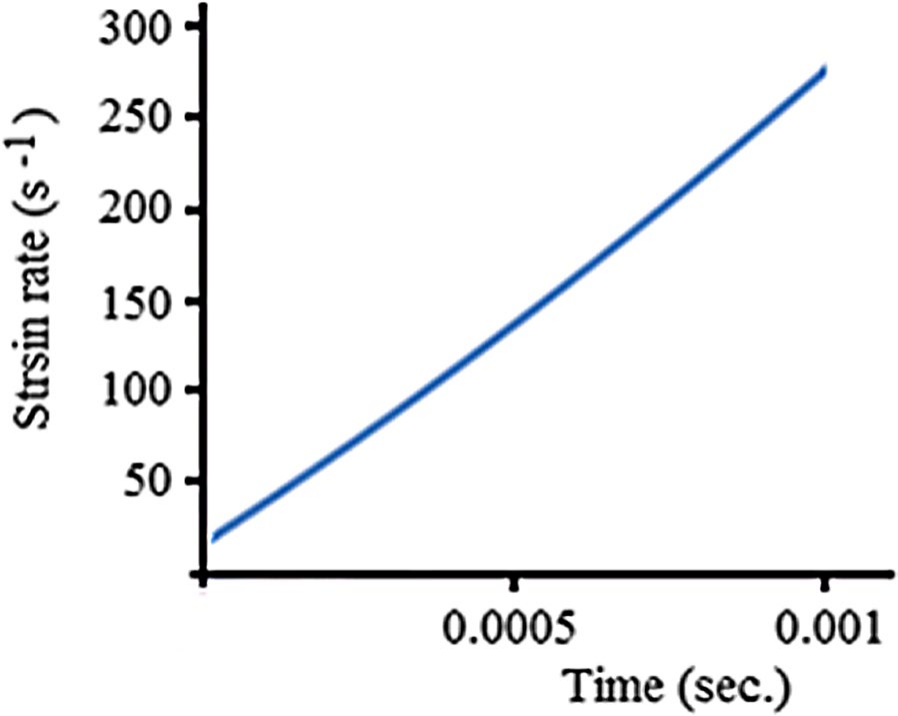
The strain rate history of the tested specimens

In order to calculate the strain rate history of the performed tests, the history of the above-mentioned mean values was treated using Eq. (1), and Fig. 15 presents the achieved results. Clearly, Fig. 15 proves the following:

- The current tester is capable of straining steel specimens at maximum rate of about 250 s^−1^, which is normal, as the typical value of the maximum strain rate for the traditional DW tester dos not exceed 300 s^−1^; see Fig. 1.
- The current tester is not capable of loading specimens at constant strain rate, which is also normal, as many well-established DW and SHB testers have the same disadvantage (Chan 2009; Borsutzki et al. 2005).

## Results comparison

**Fig. 16:**
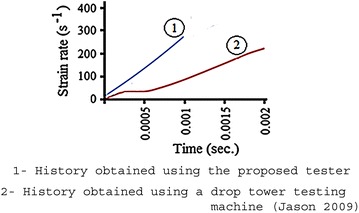
Comparison of strain rate histories

In order to evaluate the performance of the proposed tester, a comparison between the history obtained using the tester and corresponding history obtained by Chan (2009), who uses an Instron drop tower testing machine and tested samples made of titanium, was accomplished. Clearly, the compared histories, as plotted in Fig. 16 are not identical mostly due to differences in material behaviors, however, they are similar if matters such as the following are addressed:

- The maximum achievable values of strain rates.
- The non-constant values of the calculated strain rates.
- The time scale of the specimens loading.

## Conclusions

The current work dealt with a new design of high strain rate tensile testing equipment. The working principle of the introduced design includes a pendulum device which delivers an impact axial load to a specially designed specimen which extents up to failure at a particular strain rate. In order to evaluate the achievable straining properties a detailed design of the proposed machine was developed, the machine was then produced, instrumented and tested. The presented data and the obtained results, as hosted in the current paper, prove the following:

1. The proposed tester can be seen as a standard impact testing equipment in which the base that supports the specimen is replaced by the three bars that the traditional SHB uses. Additionally, the new design can also be seen as a modified form of the DW tensile tester.
2. The new tensile testing equipment is capable of straining steel specimens up to failure at maximum rate of 250 s^−1^.
3. The maximum achievable strain rate of the new tester is comparable to that of the DW testers.
4. The straining performance of the new testing equipment is not constant as it increases almost linearly when testing specimens made of structural steel.
5. The non-constant straining behavior of the tester is typical when compared with those of well-known high strain rate testing technologies.
6. The performance of the new equipment is considerably adequate, as the maximal value of the calculated standard deviations did not exceed twice the nominal value of the corresponding standard deviation (Barford 1987).
